# Exploring immune activation patterns in HER2-low and HER2-ultralow breast cancer subtypes

**DOI:** 10.1093/oncolo/oyaf081

**Published:** 2025-06-23

**Authors:** Gyöngyi Munkácsy, Libero Santarpia, Balázs Győrffy

**Affiliations:** National Laboratory for Drug Research and Development, 1117 Budapest, Hungary; Department of Bioinformatics, Semmelweis University, 1094 Budapest, Hungary; Pfizer AG, 6300 Zug, Switzerland; Department of Bioinformatics, Semmelweis University, 1094 Budapest, Hungary; HUN-REN, Research Centre for Natural Sciences, Institute of Molecular Life Sciences, Cancer Biomarker Research Group, H-1117 Budapest, Hungary; Department of Biophysics, Medical School, University of Pecs, H-7624 Pécs, Hungary

**Keywords:** ERBB2, biomarker, gene expression, anti-HER2 therapies, immune landscape, HER2-ultralow

## Abstract

**Background:**

A deeper understanding of the molecular and clinical characteristics of HER2-low and ultralow breast cancer (BC) subtypes is essential for advancing therapeutic strategies.

**Methods:**

Three independent GEO datasets with microarray and IHC/FISH data from 510 BC patients were analyzed to establish reliable HER2 expression cutoff values (>3034 for HER2-positivity and <1780 for HER2-ultralow), defining HER2-positive (HER2+), HER2-low, and HER2-ultralow cohorts. Combined with hormone receptor status, six distinct BC subgroups were identified. Prognosis was evaluated using univariate and multivariate survival analysis in a dataset of 7830 BC patients, alongside correlative analysis of 17 immune-related gene signatures across subgroups. A PubMed literature review compared our findings with existing studies.

**Results:**

In hormone receptor-positive (HR+) patients, HER2-low tumors were associated with better prognosis than HER2-ultralow and HER2+ subgroups (*P* = .0048 for relapse-free survival (RFS) and *P* = .0015 for distant-metastasis-free survival (DMFS)). No prognostic significance was observed in HR-negative (HR−) patients. Immune gene activation was consistently higher in HR− tumors, with HER2-low (HR+ and HR−) and HR-/HER2+ patients showing significant immune signature overlap. While HR+/HER2-ultralow and HR+/HER2+ patients had modest immune activation, HR-/HER2-ultralow patients exhibited the strongest association with immune signaling, including IFN signaling, T cell-activating cytokines, and cytotoxic effector molecules.

**Conclusions:**

These findings, supported by a comprehensive literature review, indicate that patients with HER2-low and HER2-ultralow BC exhibit distinct immune patterns, which supports their classification as unique BC subgroups.

**Implications for Practice**

This study underscores the significance of differentiating between HER2-low and HER2-ultralow breast cancer (BC) subtypes. In patients with hormone receptor-positive BC, HER2-low status is correlated with a more favorable prognosis compared to those classified as HER2-ultralow or HER2-positive. Furthermore, distinct patterns of immune activation were identified, particularly in hormone receptor-negative, HER2-ultralow cases. Although these findings are encouraging, further clinical validation is required before they can be implemented into standard medical practice. By conducting a comprehensive literature review on this subject, this research provides a valuable summary that may inform future clinical investigations.

## Introduction

Breast cancer (BC) is the most commonly diagnosed cancer type and the most prevalent among women globally, with a recent increase observed in younger women. According to predictive models, the burden of BC is expected to grow significantly in the next decade.^1^ Breast cancer has been classified into four main different subtypes from an immunohistochemical perspective based on the expression of hormone receptors (HRs) and human epidermal growth factor (HER2) (namely luminal A, luminal B, HER2-positive, and triple-negative),^2^ and such classification still plays a key role in determining prognosis and treatment strategies.^3-5^ Of these BC subtypes, patients with HER2-positive account for about 20%, where cancer cells overexpress HER2 protein, leading to aggressive tumor growth.^6^ Immunohistochemistry (IHC) and *in situ* hybridization (ISH) technology are used to determine the HER2 molecular subtype classification,^7-10^ and the results of these molecular tests are widely used for treatment decisions.

Traditionally, HER2 status has been used for binary classification of HER2-positive (HER2+) and HER2-negative (HER2−)^11,12^ BC, respectively, with the development of treatment strategies largely driven by such categories. Recent reports have categorized patients with HER2− BC into HER2-low and HER2-ultralow categories. HER2-low tumors have an IHC score of 1 or 2 for HER2 expression, but no evidence of HER2 amplification on molecular ISH test (ISH = negative),^13,14^ while patients with HER2-ultralow BC show ≤10% of cancer cells with faint or incomplete membrane staining and IHC score of 0.^15^ Another important approach for targeted therapeutic strategy in BC is determined by hormone receptor status. Hormone receptor-positive (HR-positive, HR+) tumors express estrogen (ER) and/or progesterone receptors, whereas hormone receptor-negative (HR-negative, HR−) tumors lack expression of both HRs.

Several clinical trials demonstrated the effectiveness of targeted therapies directed at HER2 and its pathway. Treatments such as trastuzumab (Herceptin) and pertuzumab (Perjeta), alone or in combination, have significantly improved outcomes for patients with early stage and metastatic HER2+ BC.^16-21^ Conversely, treating the HER2-/HR− subgroup, including patients with HER2-low and HER2-ultralow BC, has remained a challenge for many years due to a lack of effective targeted therapies.^22,23^ However, recent studies have demonstrated the clinical efficacy of various antibody-drug conjugates (ADCs) for these tumor types.^24-26^ The recent approach using ADCs may enable the extension of potent anti-HER2 therapy into HER2-low and HER2-ultralow BC.^27,28^

Despite significant advancements in understanding the biology of patients with HER2-low and HER2-ultralow BC and the promising potential of ADCs, heterogeneous findings, and clinical outcomes across studies underscore the need for a comprehensive summary of existing research. In this study, we examined the correlation between HER2 protein and gene expression, categorizing patients into three main groups based on HER2 levels of expression: HER2-positive, HER2-low, and HER2-ultralow. Patients with HER2-zero BC (IHC = 0, ISH = 0) were excluded from the analysis. Then the prognosis of 7,830 BC patients was evaluated within the subgroups identified. Our analysis revealed specific immune-related gene signatures for different HER2 subgroups. Lastly, we reviewed literature to find clinical data supporting our results.

## Methods

### Identification of datasets with HER2 status

A comprehensive search was conducted in GEO using the terms “breast cancer,” “erbb2,” “gpl570,” and “gpl96” to find publicly available BC datasets with both gene expression data and known HER2 status. The dataset selection and analysis only included patients whose tumor data specified HER2 status determined by fluorescence FISH or IHC, as well as gene-expression-based determination of ERBB2 levels.

HER2-positive BC is defined as tumors with an IHC score of 3+ or 2+ with HER2 amplification confirmed by FISH. HER2-low tumors have an IHC score of 1+ or 2+, without HER2 amplification (FISH-negative). HER2-ultralow BC shows an IHC score of 0, but with faint or incomplete membrane staining in ≤10% of tumor cells, differing from completely HER2-negative cases (IHC 0, no staining). The GPL570 and GPL96 platforms were chosen because they measure the same genes using identical probe sequences.

**Figure 1.**
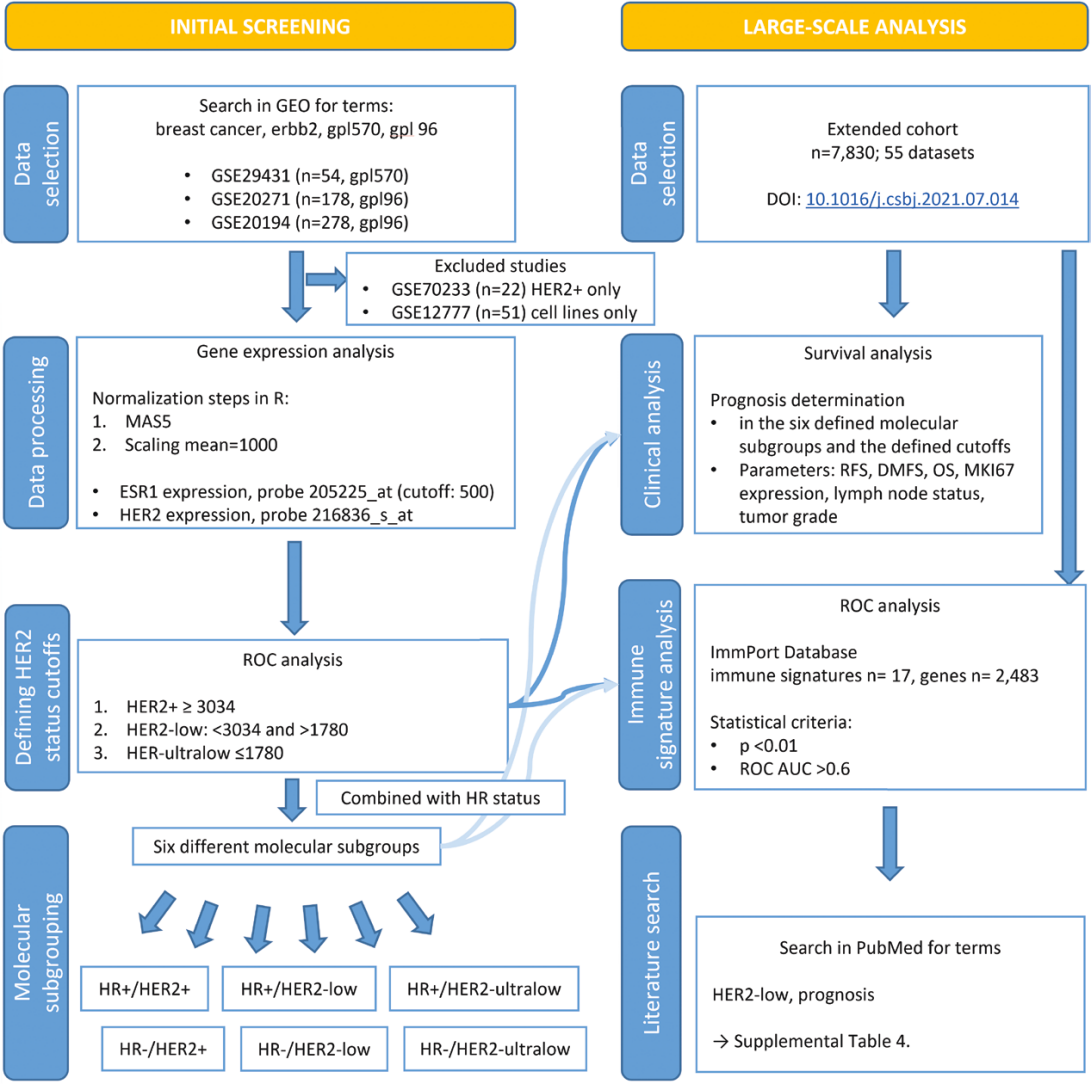
Study workflow illustrating the analytical pipeline for characterizing immune signatures in patients with HER2-low and HER2-ultralow BC. The workflow consists of initial screening of GEO datasets, data processing including normalization and gene expression analysis, definition of molecular subgroups based on HER2 and HR status, and subsequent large-scale analysis including survival outcomes and immune signature evaluation. Finally, a literature search was conducted in the PubMed database to identify publications consistent with our results.

A total of five datasets met the search criteria. However, two out of these datasets were excluded from further analysis. The GSE70233 dataset was omitted because it included only patients with HER2+ BC (*n* = 22), making it unsuitable for classification purposes. Additionally, the GSE12777 dataset was excluded as it comprised solely cell line-based gene expression data (*n* = 51). Data selection process and all analytical steps performed in this study are illustrated in Figure 1.

### Data processing for gene expression measurements

The analysis was executed in the R statistical environment (https://www.r-project.org/). A two-step normalization was made: first MAS5 and then a scaling to set the mean expression in each sample to 1000 to reduce batch effects. The used R packages include affy^29^ and simpleaffy.^30^ To determine ESR1 expression for ER status, we used the probe set “205225_at” (cutoff = 500) and for HER2 expression, we employed the probe set “216836_s_at”.

### Stratification of BC molecular subgroups

This study focused on patients with HER2-low and HER2-ultralow subtypes, new classifications within the HER2-negative spectrum. In the first analysis, we established a cutoff value to define these groups (HER2-low and ultralow groups) using the receiver operating characteristic (ROC) analysis comparing HER2 expression (HER2 expression as a continuous variable) in patients with HER2+ vs HER2-low BC, and HER2-low vs HER2-ultralow BC. Analysis of HR and HER2 levels identified six molecular BC subgroups: HR+/HER2+, HR+/HER2-low, HR+/HER2-ultralow, HR−/HER2+, HR-/HER2-low, and HR-/HER2-ultralow.

### BC datasets for survival and immune-score analysis

An integrated database with data from 55 independent BC datasets, including clinical data, was used.^31^ This database included 7830 BC patients, which were categorized into six subgroups based HER2 and HR expression levels. The cutoff values determined from the data of 510 patients were applied to this larger cohort, as ROC analysis showed these thresholds were reproducible across different datasets. Subsequent analysis was conducted using all available samples within these subgroups separately.

### Statistical analysis

The prognosis was evaluated using the following clinical endpoints: relapse-free survival (RFS), distant-metastasis-free survival (DMFS), and overall survival (OS). The analysis also considered clinical parameters such as MKI67 expression, LN status, and tumor grade. The expression of 17 immune signatures, representing various immune genes and related activated pathways, was evaluated in the six defined molecular BC subgroups. These immune signatures were derived from ImmPort (Immunology Database and Analysis Portal), a curated database supported by the National Institute of Allergy and Infectious Diseases (https://www.immport.org). Gene extraction from the database is described in detail previously.^32^ A gene was accepted as significant with a *P*-value below .01 and an ROC area under the curve (AUC) value over 0.6.

### Literature search

To contextualize our findings within the broader landscape of HER2-low and HER2-ultralow research, we performed a systematic review of previous studies. A PubMed search was conducted using the terms “HER2-low” and “prognosis.” For each identified study, we summarized the key objectives, methods, and conclusions, along with demographic, pathological, and clinical parameters. Furthermore, we indicated whether the findings aligned or conflicted with our results in the identified BC subgroups.

## Results

### Determination of HER2-low and HER2-ultralow cohorts

**Figure 2.**
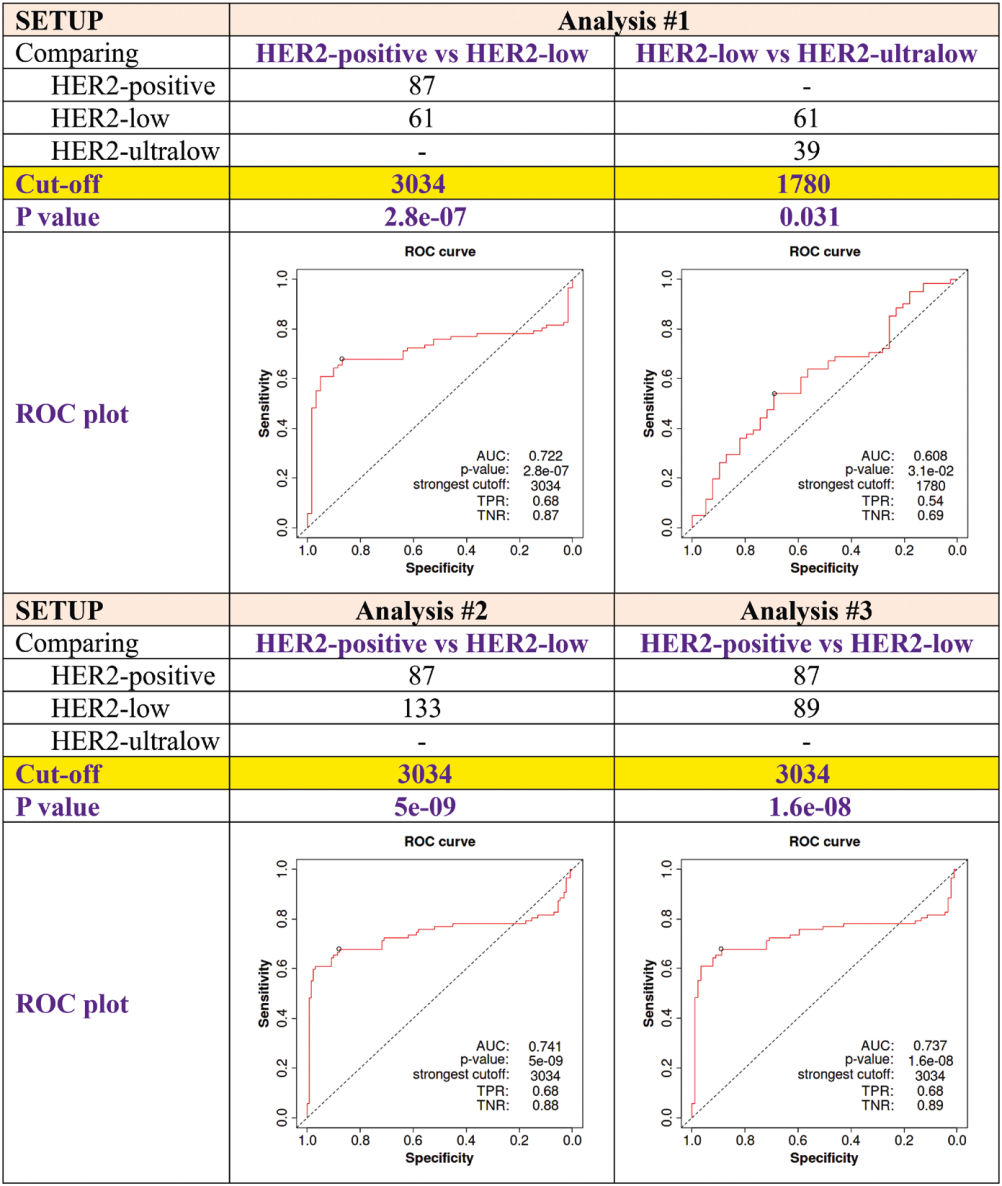
HER2 cutoff values and ROC plots in each of the analysis settings. This figure presents the ROC curves for three separate analyses (Analysis #1, #2, and #3) comparing the performance of a diagnostic test with binary classification of different outcomes. Each analysis provides the values for the compared subgroups, as well as the calculated cutoff thresholds and corresponding *P*-values. AUC values are also reported for each analysis, indicating the overall discriminative ability of the test. Abbreviations: AUC, area under the curve; ROC, receiver operating characteristic; TPR, true positive rate; TNR, true negative rate.

We analyzed BC tumor samples from 510 patients, with HER2 gene expression quantified by microarrays and HER2 status determined through IHC and FISH. Using this data, we established cutoff values to classify the patients into HER2+, HER2-low, and HER2-ultralow cohorts. The 510 patients were derived from three independent datasets. (1) GSE29431 contained 54 patients with HER2 gene expression measured using GPL570 arrays; (2) GSE20271 comprised 178 samples with gene expression obtained using GPL96 chips; (3) GSE20194 had a total of 278 patients with gene expression analyzed using GPL96 arrays—these sum to a total of 510 patients. We have performed three settings for the IHC/FISH-based HER2 status, first, we compared patients with HER2+, HER2-low, and HER2-ultralow BC (Table S1A). Second, we compared HER2+ vs HER2-low BC patients (Table S1B). Third, we compared patients with HER2+ vs HER2-low, including patients with a FISH < 2 (Table S1C). When analyzing the cohorts independently, an identical cutoff value was recognized in each of the three cohorts. Figure 2 shows the cutoff values and the ROC plots. The cutoff value for HER2 expression (≥3034) resulted in consistent HER2 positivity across all three investigated cohorts, indicating its reliability across different databases.

### BC database setup

**Figure 3.**
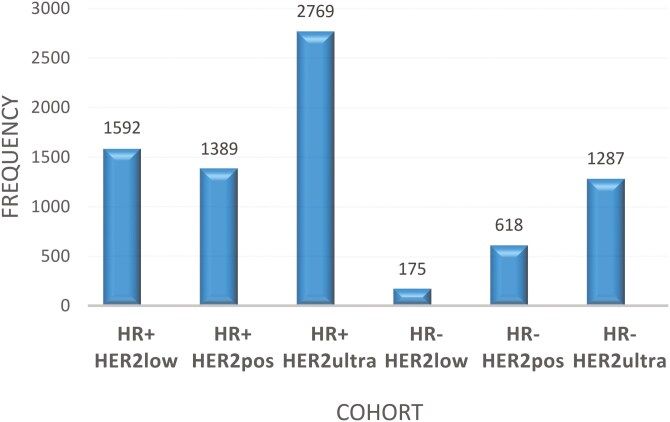
Distribution and prevalence of all investigated samples across the six molecular BC subgroups. The *x*-axis represents the various cohorts, including HR+ HER2low, HR+ HER2pos, HR+ HER2ultra, HR− HER2low, HR- HER2pos, and HR− HER2ultra. The *y*-axis shows the frequency or count of each cohort. The graph visually demonstrates the varying frequencies of the different cohorts, with the HER2ultra cohort having the highest frequency at 2769, followed by the HR+ HER2low cohort at 1592, and the HR− HER2ultra cohort at 1287. The other cohorts have relatively lower frequencies.

A specific cutoff for HER2 expression levels was determined >3034 for HER2 positivity and <1780 for HER2-ultralow. This cutoff identified six distinct HER2 BC molecular subgroups. In the integrated database of 7830 patients, irrespective of HR positivity, the distribution of patients with HER2-low and HER2-ultralow BC was 22.5% and 52%, respectively. The distribution of patients across the six molecular subgroups in the entire database is shown in Figure 3. Clinicopathological features, including age, ER status, HER2 status, lymph node (LN) status, grade, treatment, RFS, and disease-free survival (DFS) data of the 7830 patients are summarized in Table S2.

### Association of the BC subgroups with clinical outcome

**Figure 4.**
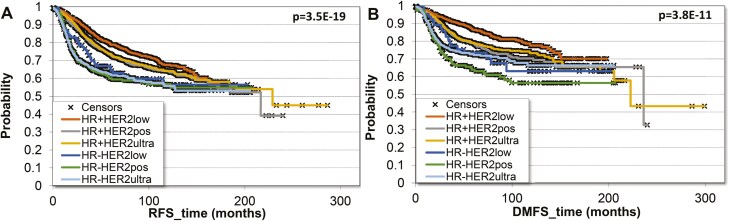
RFS and DMFS among the patients with different molecular BC. Kaplan–Meier plots showing the probability of RFS (A) and DMFS (B) over time for different cancer cohorts. The different lines correspond to various cohorts, including HR+ HER2low, HR+ HER2pos, HR+ HER2ultra, HR− HER2low, HR− HER2pos, and HR− HER2ultra.

In patients with HR+BC, HER2-low status showed a better prognosis as compared to the patients with HER2-ultralow and HER2+ BC (RFS and DMFS *P* = .0048 and *P* = .0015, respectively), while there was no prognostic effect of HER2 expression in the HR− subgroups. Not surprisingly, an association with higher grades was demonstrated in all patients with HR− tumor as compared to the HR+ subgroups regardless of HER2 status. Kaplan–Meier survival plots are presented in Figure 4. As anticipated, elevated tumor grade was observed across all patients with HR- BC compared to HR + subgroups, irrespective of HER2 status.

### Expression of immune-related genes in the defined BC subgroups

**Figure 5.**
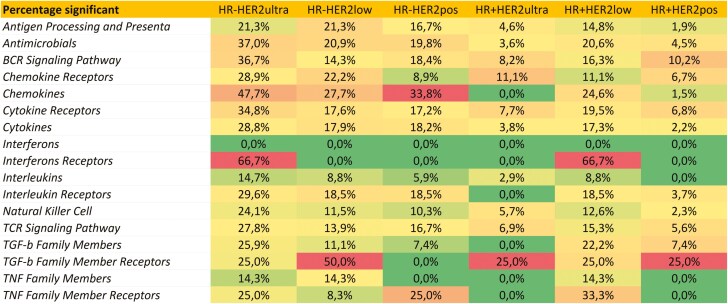
Percentage of immune genes significantly associated with the activation of immune signaling in each of the six molecular subgroups. This figure displays the percentage of significantly associated genes from distinct immune-related groups for each of the six molecular subgroups. Cells colored red indicate a higher proportion of genes from the immune-related group that are significantly associated with that subgroup. Conversely, greener cells indicate a smaller proportion of such genes. The color coding is adjusted separately for each subgroup.

The complete lists of all immune genes are provided in Table S3. Significant associations (*P* < .05, AUC > 0.6) with immune signatures were noted in various subgroups, whether positive or negative. Overall, HR- subgroups had more immune gene involvement than HR+ subgroups. Notably, HER2-low (HR+ and HR−) and HR-/HER2+ groups shared 71% of immune signature expressions meeting both criteria for significance (*P* < .05 and AUC > 0.6). The HR+/HER2-ultralow and HR+/HER2 + subgroups exhibited minimal activation of immune pathways, whereas the HR-/HER2-ultralow subgroup demonstrated significant activation of immune-related genes. In this subgroup, a substantial proportion of immune genes were activated across various immune categories. There was an activation in genes related to immune signaling pathways, including interferon receptors (67% of genes with altered expression), T cell active cytokine receptors (34% of genes), and cytotoxic effector molecules (48% of genes). Figure 5 displays the percentage of significant genes in each of molecular subgroup.

### Literature search

We reviewed previous studies downloaded from the PubMed database on HER2 classification, highlighting differences in the clinical and molecular interpretations of the HER2-low and HER2-ultralow categories. To ensure reliable data for this overview, we excluded publications prior to 2021. Among the 65 studies that met the search criteria, the majority (*n* = 46) focused on primary tumors. Most of these results align with our data, demonstrating that a distinct HER2-low subgroup exists beyond the standard HER2 classification and can be used in clinical settings. However, data on metastatic patients is insufficient and may reveal additional insights. Table S4 summarizes detailed patient data and clinical outcomes.

## Discussion

HER2-low and HER2-ultralow BC subtypes fall outside the traditional binary classification of HER2 tumors, posing challenges in predicting their response to standard anti-HER2 therapies. In this study, we aimed to stratify BC patients based on the HER2 status using gene expression data and evaluate prognosis and immune correlates across different HER2 subtypes. For this, we analyzed data from three independent GEO datasets, which included microarray and IHC/FISH data from 510 BC patients, to establish reliable HER2 expression cutoffs. The HER2-zero subgroup was excluded from this study to ensure a focused analysis of the distinct molecular profiles of HER2-low and HER2-ultralow tumors, which are defined by low-level HER2 expression. Using these cutoff values, patients with HER2 classification were divided into six molecular subgroups, based on HER2+, HER2-low, and HER2-ultralow features combined with two HR statuses. Finally, an immunological analysis was conducted, and a literature search was performed.

Our findings regarding the prognostic significance of HER2 classification indicated that patients with HER2-low BC, particularly within the HR+ subgroup, exhibited a more favorable prognosis compared to those with HER2-ultralow and HER2+ tumors. Consistent with these results, a study of 3,512 BC patients from four neoadjuvant clinical trials found significant clinical and pathological differences between patients with HER2-low and HER2-zero (IHC = 0) tumors. Patients with HER2-low BC had fewer grade III statuses, lower proliferation (Ki67>35), and fewer TP53 mutations compared to those with HER2-zero tumors.^33^ Additionally, patients with HER2-low BC exhibited a reduced pathologic complete response (pCR) rate, particularly in patients with HR+ status. As noted in Table S4, various studies have reported differing prognoses across HER2 subtypes. This variation may be attributed to a lack of standardized diagnostic methods ensuring reliability and reproducibility, as well as differences in the samples and data analyzed. Our findings within this context suggest that immunological stratification of BC based on HER2 expression levels may offer additional insights beyond the traditional binary classification. HER2 and its expression levels may affect the tumor microenvironment and immune cells contextures; however, their impact on the efficacy of immunotherapies remains an area of active investigation. Our data demonstrated that overall immune gene activation was higher in patients with HR− BC compared to those with HR+ status, with an overlap observed between certain immune signatures HER2-low (HR+ and HR−) and HR−/HER2+ subgroups. Of note, BC patients in the HR-/HER2-ultralow subgroup had the most significant activation of immune signaling pathways, mainly represented by an immune suppression environment. Recent studies suggest that group of patients with HER2-ultralow BC is a highly heterogeneous group with a distinct biological profile and immune profile affecting patients’ outcomes and response to therapies.^34^ The varying tumor-immune profiles in BC subgroups indicate that targeted immunotherapy could be beneficial for certain patients, though these findings are still exploratory.

Researchers are currently investigating therapeutic options for these tumors including immunotherapy and other new strategies. A phase II study of atezolizumab and nab-paclitaxel as part of neoadjuvant therapy in patients with early stage TNBC showed promising activity in this high-risk population. In this study, pCR was significantly higher for patients with HER2-negative (IHC 0) vs HER2-low tumors (OR 1.73, *P* = .036).^35^ A recent study examined immune-related gene signatures in HER2-negative BC (TNBC) patients treated with immune checkpoint inhibitors.^36^ It found that tumors with ER levels of 1%-9% and 10%-50% had similar levels of stromal TILs, CD8+ T cells, and PD-L1 positivity to ER 0% tumors, but higher levels than those with ER ≥51%. In a similar study comprising early stage BC patients, most clinicopathological features did not differ between patients with HER2-low and HER2-negative BC. However, after adjusting for different clinical variables, patients with HER2-low status had worse DFS than patients with HER2-negative tumors^37^ These data indirectly support our results suggesting that the immune system of ER-low and –intermediate BC statuses of tumors behave similarly to that of primary TNBC.^36^

HER2-targeted therapies, such as ADCs, both as standalone treatments and in combination with other therapies, can have important immune-mediated effects in metastatic BC. The ADC trastuzumab deruxtecan (T-DXd) has shown the most significant activity in HER2-low metastatic BC, with a 37% response rate and 11-month PFS in heavily pretreated patients.^38^ T-DXd also demonstrated longer PFS and OS compared to the physician’s choice of chemotherapy.^39^ In the phase 3 DESTINY-Breast04 and DESTINY-Breast06 trials T-DXd significantly improved PFS in HR+/HER2-low and HR+/HER2-ultralow advanced BC patients. Disitamab vedotin, an ADC targeting HER2, has shown clinical efficacy in HER2+ and HER2-low BC, both as a single agent and in combination with other therapies.^40^ Ongoing trials are evaluating its effectiveness in patients with advanced HER2-low and metastatic HR+ BC. Combinations of ADC with immune checkpoint inhibitors, such as nivolumab and T-DXd have shown an overall response rate (ORR) of 38% and a PFS of 6.3 months in patients with advanced HER2-low BC.^41^ Durvalumab in combination with T-DXd showed promising early safety and efficacy results in first-line HER2-low-expressing triple-negative BC (TNBC) patients with a 66.7% ORR, irrespective of the PD-L1 expression.^42^ Although chemotherapy remains the primary treatment for patients with TNBC, pembrolizumab has been approved for use in combination with chemotherapy for patients with advanced metastatic TNBC. In a multicenter study, more than 400 HR+/HER2- advanced BC patients received first-line endocrine therapy combined with CDK4/6 inhibitors. Among them, HER2-low status was associated with worse PFS and OS.^43^ In a US study of over 1 million patients, HER2-low status was linked to slightly better survival particularly in advanced TNBC, with a 2% increase in 5-year OS. A slight reduction in pCR was observed in patients with HER2-low disease undergoing neoadjuvant chemotherapy.^44^

We must address several important methodological issues in this study. First, the cutoff values for HER2 expression were reproducible across multiple datasets, demonstrating the robustness and consistency of our method. However, as the analysis was limited to primary tumor data and conducted retrospectively, further validation in independent patient cohorts is necessary. Another limitation is the suboptimal AUC value of 0.608 for the HER2-ultralow cutoff. Generally, event with a statistical significance, AUC values below 0.6 are considered to have low discriminatory power and are not regarded as clinically valuable.^45^ Despite the statistically significant differences between the HER2-low and HER2-ultralow categories, the modest AUC warrants caution in interpreting the biological significance.

Accurately identifying patients with HER2-low and HER2-ultralow BC is becoming essential for selecting appropriate treatment strategies and improving patient outcomes. Several diagnostic strategies are being developed, and our approach uses mRNA expression levels to define additional BC molecular subgroups. In support of our methodology, different studies have demonstrated a direct and strong correlation between ERRB2 mRNA and protein expression levels^46,47^ offering the advantage of reliable assessment of HER2. A study involving 749 patients showed a 91% concordance for HER2 status between microarray and local IHC/FISH assessments^48^ and 93% in the MINDACT trial.^49^ Emerging transcriptomic techniques, such as RNA sequencing, accurately classify HER2 status and can assist in guiding treatment decisions, particularly for HER2-targeted therapies.^50^ Assessing dynamic HER2 expression could help enhance treatment strategies, determine the optimal timing for changing cancer therapies, and identify patients who may qualify for new clinical trials. While RNA-based methods show potential in correlating with traditional HER2 testing, the current gold standards for clinical HER2 classification remain IHC and FISH.

## Conclusion

Accurately identifying HER2-low and HER2-ultralow statuses in BC patients is becoming increasingly important for developing better treatment strategies. While recent findings indicate that HER2 expression levels may influence interactions with the tumor and immune microenvironment, these immune correlates represent only one aspect of the complex biology of these subtypes. The full biological profile of HER2-low and HER2-ultralow tumors remains to be fully characterized. Antibody-drug conjugates or immunotherapies could be potential options for patients with immune activation signatures, but these therapies require further clinical evaluation. Future research and clinical trials that incorporate comprehensive biological assessments of HER2 expression changes are necessary to optimize treatment strategies for patients with HER2-low and HER2-ultralow BC.

## Supplementary Material

oyaf081_suppl_Supplementary_Tables_1

oyaf081_suppl_Supplementary_Tables_2

oyaf081_suppl_Supplementary_Tables_3

oyaf081_suppl_Supplementary_Tables_4

## Data Availability

All data generated or analyzed during this study are included in this published article and its supplementary information file.
