# Supplementary material for: Exploring immune activation patterns in HER2-low and HER2-ultralow breast cancer subtypes
Source: Oncologist. 2025 Jun 23;30(6):oyaf081. doi: 10.1093/oncolo/oyaf081 (PMC12205994; doi:10.1093/oncolo/oyaf081)
Supplement: oyaf081_suppl_Supplementary_Tables_1 [file oyaf081_suppl_supplementary_tables_1.docx]

**Supplemental Table 1.** Definition of HER2 cohorts based on FISH (yellow) and IHC (green) values assigned to each patient in the initial database. The first row in each blue cell indicates the classification criteria used for cut-off determination in patients with (A) HER2+, HER2-low, and HER2-ultralow breast cancer; (B) HER2+ and HER2-low breast cancer; and (C) HER2+ and HER2-low breast cancer excluding IHC=0 cases. The second row in each blue cell provides the number of patients meeting the specified criteria.
