# Supplementary material for: Exploring immune activation patterns in HER2-low and HER2-ultralow breast cancer subtypes
Source: Oncologist. 2025 Jun 23;30(6):oyaf081. doi: 10.1093/oncolo/oyaf081 (PMC12205994; doi:10.1093/oncolo/oyaf081)
Supplement: oyaf081_suppl_Supplementary_Tables_2 [file oyaf081_suppl_supplementary_tables_2.docx]

**Supplemental Table 2. Clinicopathological characteristics of patients (n=7,830).** The patients were obtained from 55 distinct Gene Expression Omnibus (GEO) Series. *Abbreviations: ACT = adjuvant chemotherapy, DFS= disease-free survival, ER = estrogen receptor, ET = endocrine therapy, HER2 = human epidermal growth factor receptor, LN = lymph node, NA = not available, NACT = neoadjuvant chemotherapy, RFS = relapse-free survival.*

| **Age** | **Mean** | 53.51 |  |
| --- | --- | --- | --- |
|  | **Median** | 52.3 |  |
|  | **Min** | 24 |  |
|  | **Max** | 93 |  |
| **ER status** | **negative** | 2080 | 26,6% |
|  | **positive** | 5750 | 73,4% |
| **HER2 status** | **negative** | 5823 | 74,4% |
|  | **positive** | 2007 | 25,6% |
| **LN status** | **0** | 2858 | 36,5% |
|  | **1** | 2212 | 28,3% |
|  | **2** | 7 | 0,1% |
|  | **3** | 5 | 0,1% |
|  | **NA** | 2748 | 35,1% |
| **Grade** | **I** | 576 | 7,4% |
|  | **II** | 1818 | 23,2% |
|  | **III** | 2111 | 27,0% |
|  | **NA** | 3325 | 42,5% |
| **Treatment** | **0** | 3821 | 48,8% |
|  | **1** | 1030 | 13,2% |
|  | **NA** | 2979 | 38,0% |
| **ACT** | **0** | 3313 | 42,3% |
|  | **1** | 925 | 11,8% |
|  | **NA** | 3592 | 45,9% |
| **ET** | **0** | 2038 | 26,0% |
|  | **1** | 1871 | 23,9% |
|  | **NA** | 3921 | 50,1% |
| **NACT** | **0** | 2633 | 33,6% |
|  | **1** | 1615 | 20,6% |
|  | **NA** | 3582 | 45,7% |
| **RFS event** | **none** | 3525 | 45,0% |
|  | **relapse** | 1520 | 19,4% |
|  | **NA** | 2785 | 35,6% |
| **DFS event** | **none** | 1487 | 19,0% |
|  | **death** | 478 | 6,1% |
|  | **NA** | 5865 | 74,9% |
| **Molecular subtype (St Gallen)** | **luminal A (ER+ HER2- MKI67-)** | 3687 | 47,1% |
|  | **luminal B** | 2063 | 26,3% |
|  | **HER2+ ER-** | 560 | 7,2% |
|  | **TNBC** | 1520 | 19,4% |
